# Functionality study of chalcone-hydroxypyridinone hybrids as tyrosinase inhibitors and influence on anti-tyrosinase activity

**DOI:** 10.1080/14756366.2020.1801669

**Published:** 2020-08-04

**Authors:** L. Ravithej Singh, Yu-Lin Chen, Yuan-Yuan Xie, Wei Xia, Xing-Wen Gong, Robert C. Hider, Tao Zhou

**Affiliations:** a School of Food Science and Biotechnology, Zhejiang Gongshang University, Hangzhou, Zhejiang, PR China; b Division of Pharmaceutical Science, King’s College London, London, UK; c College of Pharmaceutical Sciences, Zhejiang University of Technology, Hangzhou, PR China

**Keywords:** Tyrosinase inhibitor, hydroxypyridinone, chalcone analogue, monophenolase, diphenolase

## Abstract

In an attempt to synthesise new tyrosinase inhibitors, we designed and synthesised a series of chalcone-hydroxypyridinone hybrids as potential tyrosinase inhibitors adopting strategic modifications of kojic acid. All the newly synthesised compounds were characterised by NMR and mass spectrometry. Initial screening of the target compounds demonstrated that compounds **1a**, **1d**, and **1n** had relatively strong inhibitory activities against tyrosinase monophenolase, with IC_50_ values of 3.07 ± 0.85, 2.25 ± 0.8 and 2.75 ± 1.19 μM, respectively. The inhibitory activity against monophenolase was 6- to 8-fold higher than that of kojic acid. Compounds **1a**, **1d**, and **1n** also showed inhibition of diphenolase, with IC_50_ values of 17.05 ± 0.07, 11.70 ± 0.03 and 19.3 ± 0.28 μM, respectively. The inhibition kinetics of diphenolase indicates that compounds **1a** and **1d** induce reversible inhibition on tyrosinase. Finally, we found that copper coordination should be one of the important inhibitory mechanism of these compounds in tyrosinase.

## 1. Introduction

Tyrosinase is a copper-containing metalloenzyme widely distributed in animals, plants, and microorganisms and is a key and rate-limiting enzyme for melanogenesis^1^. It is isolated from diverse resources and its structural properties are well documented^2^. A di-copper centre is the common structural unit of the active site which is found in all species. Each copper ion is coordinated by three histidine residues. Further, antiferromagnetic coupling of the two copper ions renders the active site EPR inactive^3^. During the tyrosinase-catalysed oxidation process, the binuclear metal centre activates an O_2_ molecule and subsequently oxidises substrates such as l-tyrosine and l-DOPA to catecholates and benzoquinones, respectively^4^. As tyrosinase plays a fundamental role in the melanogenesis process, the abnormal function of this enzyme poses challenges in a range of socio-economic areas such as agriculture, food, and the pharmaceutical industry^5^. Tyrosinase regulates pigmentation, and abnormal expression can lead to various pigmented diseases such as freckles and age spots^6^. If the expression is insufficient, skin diseases such as those associated with albinism and vitiligo will occur. In fruits and vegetables, tyrosinase causes browning during post-harvest processing, transportation, and storage, affecting its quality and commercial value^7^.

There are many tyrosinase inhibitors originating from both natural sources and chemical synthesis^8^. Kojic acid, vitamin C, cysteine, and arbutin are inhibitors isolated from natural sources. Although ideal in many respects, kojic acid has not found wide application due to its metabolic instability^9^. Benzaldehyde, 4-halo-benzoic acids, and 4-chlorosalicylic acid are tyrosinase inhibitors of synthetic origin^10^. In addition, extracts from animals and fish, collagen peptides, sorghum peptides have also been found to possess inhibitory effects on tyrosinase^11^^,^^12^.

Development of useful tyrosinase inhibitors, possessing potent commercial utility has been limited to date due to poor solubility, low shelf-life, and safety concerns^13^. Kojic acid, a secondary metabolite produced by Aspergillus and Penicillium moulds, provides a promising starting point for the synthesis of new tyrosinase inhibitors; the α-hydroxy ketone functionality plays an important role in tyrosinase inhibition^14^^,^^15^. Based on previous results, relating to hydroxypyridinones^16–19^, we have functionalised kojic acid to form new derivatives in attempt to identify more potent tyrosinase inhibitors.

**Figure 1.**
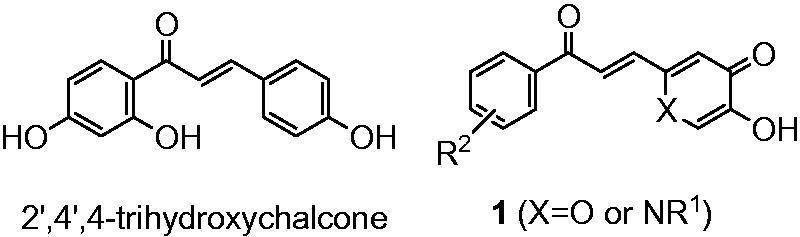
Structure of a typical chalcone and designed molecules **1**.

As many chalcones possess tyrosinase inhibitory activities (IC_50_ value for tyrosinase inhibition of 2′,4′,4-trihydroxychalcone is 8.1 μM)^20^^,^^21^, it was decided to investigate the properties of chalcone analogues (**1**) which contain either a pyranone or pyridinone ring (Figure 1).

## 2. Materials and methods

### 2.1. Chemistry

^1^H NMR and ^13 ^C NMR spectra were recorded on a Bruker Avance 400 spectrometer (Bruker Corp., Karlsruhe, Germany) with TMS as an internal standard. Electrospray ionisation (ESI) mass spectra were obtained by infusing samples into an LCQ Deca XP ion trap instrument (ThermoFinnigan, SanJose, CA). High resolution mass spectra (HRMS) were determined on Waters QTOF micro (Waters, USA). Lyophilised powder of mushroom tyrosinase (≥1000 U/mg) was purchased from Sigma. Kojic acid, l-tyrosine, and l-DOPA of analytical grade were purchased from Aladdin chemicals, Shanghai, China. All other chemicals were of AR grade and used without any further purification. 5-Benzyloxy-2-hydroxymethyl-pyran-4-one was prepared according to previous report^22^. Compounds **3a–3d**, **4a–4e**, **6a–6d** and **7a–7d** were prepared according to previous reports^16^^,^^17^^,^^23^^,^^24^. General procedure for the synthesis of compounds **1a–1o** and their physical and spectroscopic data are presented in Supplementary Materials.

### 2.2. Tyrosinase inhibition assay

#### 2.2.1. Monophenolase and diphenolase inhibition assay

Monophenolase and diphenolase activity was measured as described previously by monitoring the dopachrome absorbance at 475 nm^17^. l-Tyrosine and l-DOPA were used as reaction substrate for monophenolase and diphenolase assays, respectively. In the reaction, l-tyrosine (50 µL, 2 mM) or l-DOPA (50 µL, 0.5 mM), 90 µL of pH 6.8 phosphate buffer and 5 µL volume of different concentrations of final compounds (**1a–1o**) in DMSO were mixed and incubated at 30 °C. Finally, 20 units of enzyme was quickly added to the reaction mixture and incubated at 30 °C for 10 min. The absorbance at 475 nm was recorded with a microplate reader. During these incubations, the final concentration of DMSO was limited to 3% by volume. The assay was carried out in triplicate and DMSO was used as control^17^.

#### 2.2.2. Inhibition kinetics on diphenolase activity of tyrosinase

The same experimental protocol described above was adapted for inhibition kinetics, with different inhibitor concentration. However the absorbance was measured at 1 min reaction intervals for twelve minutes^16^.

#### 2.2.3. Reversibility of inhibitory effect of compounds on tyrosinase

Measurement method was substantially the same to that described above, changing the added concentration of the enzyme solution, different concentrations of inhibitor on mushroom tyrosinase l-DOPA as reaction substrate^16^.

### 2.3. pK_a_ and copper(II) stability constants determination

An automated titration system used in the study consists of a Metrohm 765 Dosimat autoburette, a Mettler Toledo MP230 pH metre with SENTEK pH electrode (P11), and an HP 8453 UV-visible spectrophotometer with a Hellem quartz flow cuvette being circulated through by a Gilson Mini-plus #3 pump—speed capability (20 ml/min). A potassium chloride electrolyte solution (0.1 M) was used to maintain the ionic strength. The temperature of the test solutions was maintained in a thermostatic jacketed titration vessel at 25 °C (± 0.1 °C) using a Fisherbrand Isotemp water bath. The pH electrodes were calibrated using GLEE^25^ with data obtained by titrating a volumetric standard HCl (0.1 M) in KCl (0.1 M) with KOH (0.1 M) under an argon gas atmosphere in the vessel. The solution under investigation was stirred vigorously during the experiment. For p*K*_a_ determinations, a cuvette path length of 10 mm was used while for metal stability constant determinations, a cuvette path length of 50 mm was used. All instruments were interfaced to a computer and controlled by an in-house programme. An automated titration adopted the following strategy: the pH of a solution was increased by 0.1 pH unit by the addition of KOH solution (0.1 M) from the autoburette. The pH readings were judged to be stable if their values varied by less than 0.01 pH unit after a set incubation period. For p*K*_a_ determinations, an incubation period of 1.5 min was adopted; for copper(II) stability constant determinations, an incubation period of 3 min was adopted. The cycle was repeated until the defined end point pH value was achieved. Titrations were carried out in the solution with molar ratio of DMSO: H_2_O being 0.2: 1 due to the solubility issue of samples and/or the corresponding copper(II) complexes. Under this condition, the pH metre readings are shifted, compared to the aqueous solution. All the titration data were analysed with the HypSpec2014 programme (http://www.hyperquad.co.uk/)^26^^,^^27^. The speciation plot and pM values were calculated with the HYSS programme^28^. Based on deferiprone titrations, pCu^2+^ at pH 7.6, denoted as pCu^2+^_7.6_, with [ligand]_total_ = 10 µM and [Metal]_total_ = 1 µM, were calculated as close approximations for pCu^2+^ at pH 7.4 in the aqueous solution. Analytical grade reagent materials were used in the preparation of all solutions.

### 2.4. Molecular docking study

Molecular docking was performed by using CDOCKER module embedded in Discovery Studio 2.5 software (Accelrys Software, Inc., San Diego, CA, USA)^17^. The X-ray crystal structure of tyrosinase from Agaricus bisporus (PDB ID: 2Y9X) was retrieved from the Protein Data Bank (http://www.rcsb.org/pdb). All crystallographic water molecules and ions were removed from the protein structure. 3 D structure of compound **1d** was generated in Chem3D Ultra 8.0, and conformations were generated by using a modified CHARMm force field. The obtained conformations were then docked into the binding site of tyrosinase. The docked conformation with the lowest energy was used for the analysis of binding mode.

### 2.5. Statistical analysis

All the experiments were performed in triplicate. The data were statistically analysed using MS-Excel and GraphPad Prism 6 software.

## 3. Results and discussion

### 3.1. Chemistry

**Scheme 1.**
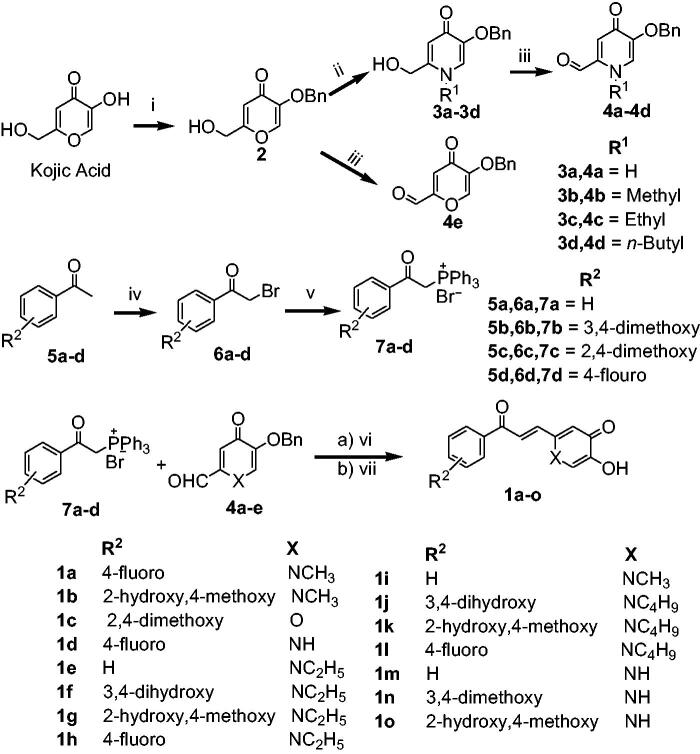
Synthetic route of compounds **1**. Reagents and conditions: (i) BnCl, NaOH, MeOH/H_2_O, 70 °C, 6 h, 80% yield; (ii) appropriate amines, EtOH, reflux, overnight, (iii) MnO_2_, 1,4-dioxane, (iv) Br_2_, CHCl_3_, rt, 10 min; (v) PPh_3_, CH_2_Cl_2_, 30 min, (vi) *tert*-BuOK, dry THF, (vii) BBr_3_, DCM, 0 °C to rt, 2 h, 55–72% yield.

The synthetic route and complete reaction conditions for the preparation of compounds **1a–1o** are illustrated in Scheme 1. The benzyl protection of kojic acid with BnCl in presence of sodium hydroxide and methanol:water (1:1) as reaction solvent at 70 °C gave the intermediate compound **2**. Further, refluxing of intermediate **2** with different aliphatic amines in ethanol gave the intermediates (**3a–3d**) in good yield. The corresponding aldehydes (**4a–4e**) were afforded by the selective oxidation of compounds **3a–3d** with active MnO_2_, in moderate yield. The phosphonium salts (**7a–7d**) were synthesised by reaction of α-bromo acetophenones (**6a–6d**) with triphenylphosphine in dichloromethane in excellent yield. Finally, the target hybrid compounds (**1a–1o**) were obtained using the Wittig reaction by the condensation of phosphonium salts **7** with aldehydes (**4a–4e**) in presence of *tert*-BuOK in dry THF and subsequent deprotection of benzyl group with BBr_3_ in dichloromethane at 0 °C. All the newly synthesised compounds were characterised using ^1^H NMR, ^13 ^C NMR and HRMS.

### 3.2. Inhibitory effect of compounds 1a–1o on monophenolase activity

**Figure 2.**
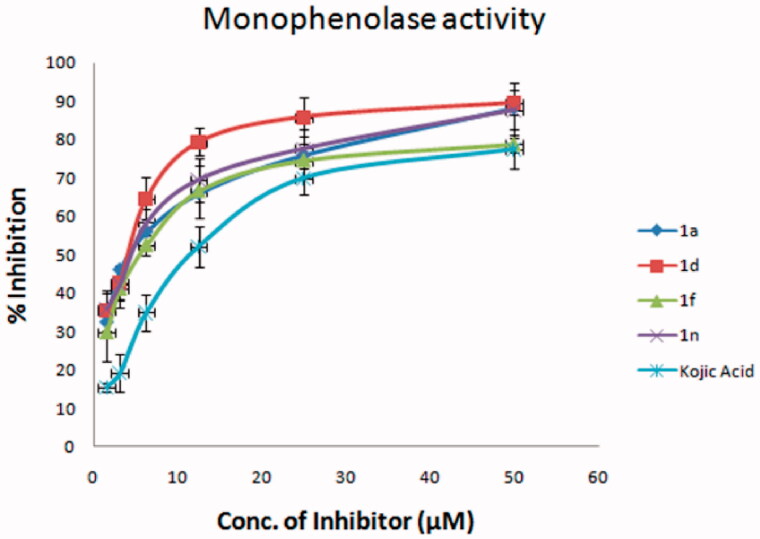
Inhibitory effect of **1a**, **1d**, **1f**, and **1n** on the monophenolase activity of mushroom tyrosinase. The assays were performed at 30 °C and pH 6.8.

All the final compounds (**1a–1o**) were screened for monophenolase inhibitory activity at 50 μM (Table 1). Four compounds (**1a, 1d, 1f,** and **1n**) were found to possess a stronger inhibitory activity on monophenolase activity of mushroom tyrosinase than kojic acid under the conditions defined in Table 1. The inhibition rate of **1a, 1d, 1f,** and **1n** was 85.3%, 89.5%, 78.8% and 87.6% at 50 μM, respectively, which was higher than that of kojic acid (75.4%). These four compounds were tested at different concentrations to realise IC_50_ values (Figure 2); **1a** 3.07 ± 0.85 μM, **1d** 2.25 ± 0.80 μM, **1f** 8.11 ± 2.67 μM, and **1n** 2.75 ± 1.19 μM. The inhibitory effects of **1a, 1d, 1f,** and **1n** were 5.7-, 7.8-, 2.2-, and 6.4-fold higher than that of kojic acid (IC_50_ = 17.55 ± 1.91 μM). Compounds **1a, 1d,** and **1n** possess obviously superior monophenylase inhibitory activity to that of 2′,4′,4-trihydroxychalcone (IC_50_ = 8.1 μM, inhibition rate is 67% at 50 μM)^20^, while the activity of **1f** is close to that of 2′,4′,4-trihydroxychalcone. The substituents on position-1 (X) and on the benzene ring (R_2_) (Scheme 1), and hydrophobicity of these molecules reflected by their calculated partition coefficients (clogP) influence the monophenylase inhibitory activity (Table 1). The four most active inhibitors possessed clogP values equal to or less than 1.6 and they also have strong electron withdrawing functions on the benzene ring, i.e. F, OH or OMe. The inductive effect will dominate for F and OMe substituents. When the pyridinone ring N is replaced by an oxygen (**1c**), low inhibitory activity resulted. When the clogP value was greater than 1.6, there was an appreciable reduction in inhibitory potency (**1c, 1 g, 1 h, 1j, 1k, 1 l**). The 2-hydroxy-4-methoxybenzene aromatic rings (**1 b, 1o**) and unsubstituted benzene (**1e, 1 m**) were also found to lack potent inhibitory properties. Thus the presence of 4-fluoro- or 3,4-dimethoxy benzene in the structure leads to optimal activity together with either a NH or NMe in the pyridinone ring.

**Table 1.** Inhibition of compounds (**1a–1o**) (50 µM) on monophenolase activity of mushroom tyrosinase under the conditions of 30 °C and pH 6.8.

Compounds	Inhibition (%)	R^2^	X	clogP^a^
**1a**	**85.3**	4-F	NCH_3_	1.60
**1b**	66.7	2-OH, 4-OCH_3_	NCH_3_	1.41
**1c**	50.3	2,4-dimethoxy	O	1.51
**1d**	**89.5**	4-F	NH	1.53
**1e**	63.3	H	NC_2_H_5_	1.81
**1f**	**78.8**	3,4-dihydroxyl	NC_2_H_5_	0.84
**1g**	71.4	2-OH, 4-OCH_3_	NC_2_H_5_	1.78
**1h**	57.8	4-F	NC_2_H_5_	1.97
**1i**	72.8	H	NCH_3_	1.43
**1j**	69.4	3,4-dihydroxyl	NC_4_H_9_	2.31
**1k**	64.6	2-OH, 4-OCH_3_	NC_4_H_9_	2.84
**1l**	50.3	4-F	NC_4_H_9_	3.03
**1m**	66.7	H	NH	1.36
**1n**	**87.6**	3,4-dimethoxy	NH	1.01
**1o**	63.3	2-OH, 4-OCH_3_	NH	1.34
Kojic acid	75.4	–	–	−0.89

^a^ The clogP values were calculated from website: http://www.molinspiration.com/cgi-bin/properties.

### 3.3. Inhibitory kinetics and reversibility on diphenolase activity of tyrosinase

**Figure 3.**
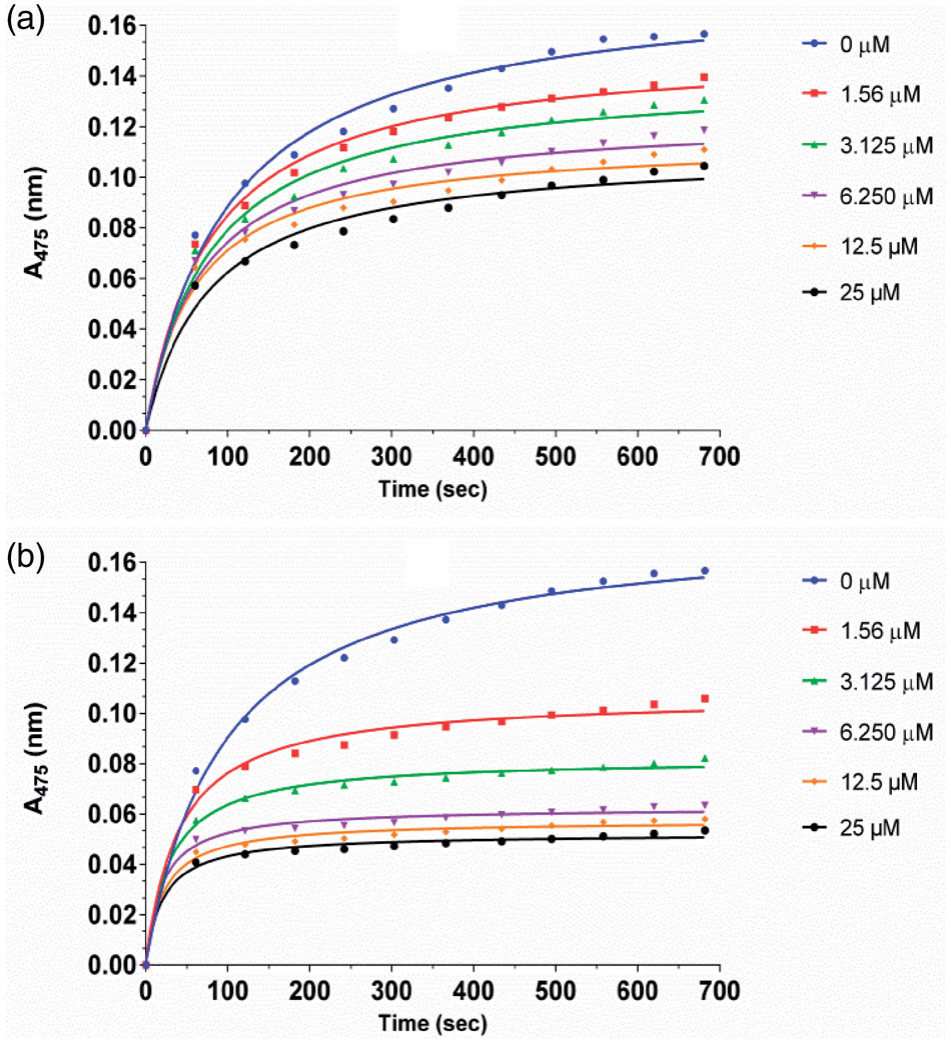
Inhibitory effect of different concentrations of **1a** (a) and **1d** (b) on the diphenolase activity of tyrosinase. The assays were performed at 30 °C and pH 6.8.

The inhibitory kinetic courses of mushroom tyrosinase in the presence of different concentrations of compounds **1a** and **1d** was investigated by monitoring their inhibition on diphenolase inhibitory activity using l-Dopa as a substrate. The formation of *o*-quinone generated by the oxidation of l-Dopa increased with time, and the absorbance values reduced with increasing concentration of compounds **1a** and **1d** (Figure 3). In addition, the reaction process catalysed by the diphenolase activity of tyrosinase had no lag time. This result is in agreement with those of previous reports^18^^,^^19^.

The inhibition of compounds **1a**, **1d,** and **1n** on the diphenolase activity of tyrosinase increased with increasing concentration of inhibitor. The IC_50_ values of **1a**, **1d,** and **1n** were determined to be 17.1 ± 0.07 µM, 11.7 ± 0.03 µM, and 19.3 ± 0.28 respectively.

**Figure 4.**
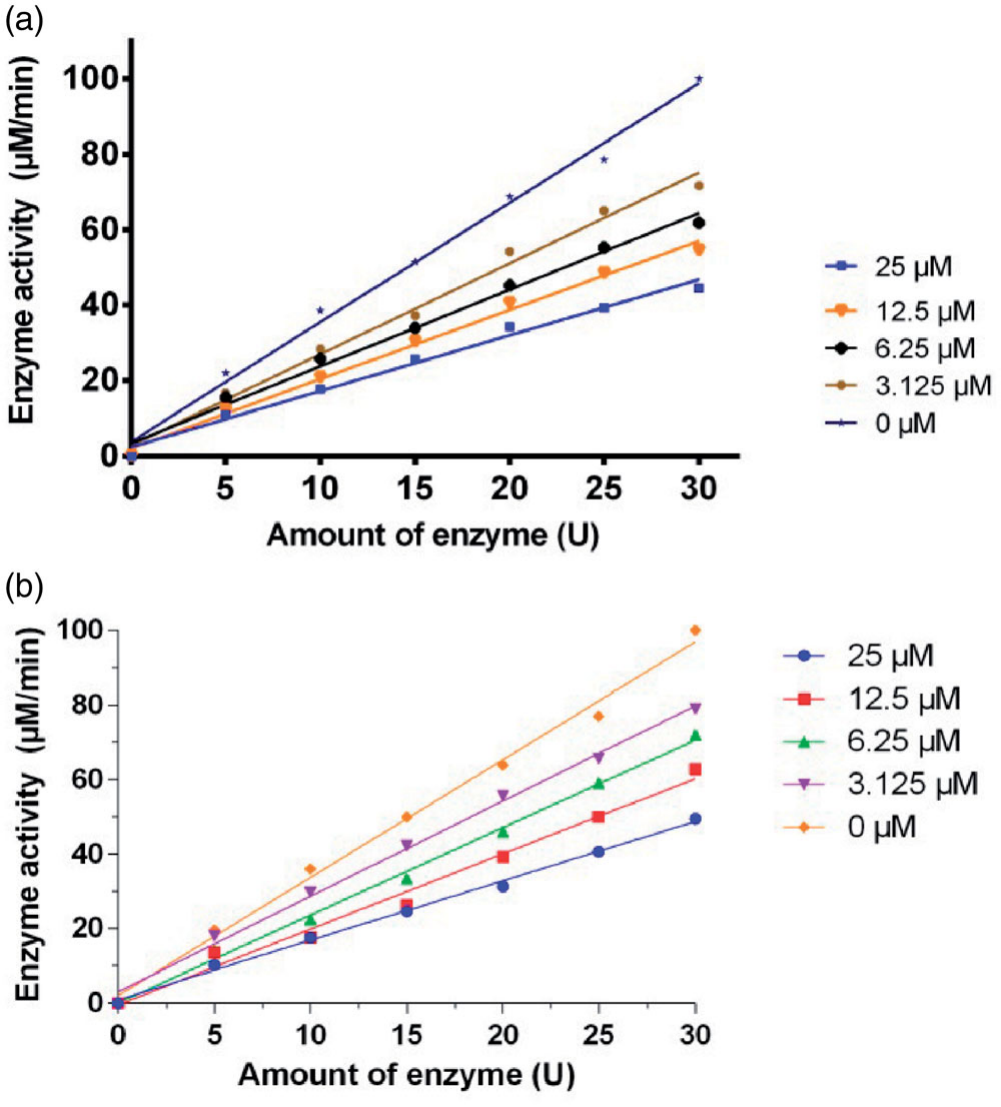
Determination of the inhibitory reversibility of **1a** (a) and **1d** (b) on mushroom tyrosinase. The concentrations of inhibitors for curves were 0.00, 3.125, 6.25, 12.5, and 25.00 µM, respectively. The assays were performed at 30 °C and pH 6.8.

The inhibitory reversibility of **1a** and **1d** on mushroom tyrosinase was investigated using l-DOPA as a substrate. For both compounds, investigation on the relationship between enzyme activity and its concentration in the presence of compounds **1a** and **1d** indicated that the plots of the remaining enzyme activity versus the concentration of enzyme at different inhibitor concentrations gave a family of straight lines, which all passed through the origin (Figure 4). Increase of inhibitor concentration resulted in descent of the slope of the line, indicating that the presence of inhibitor resulted in the inhibition of enzyme activity. Thus, the inhibition of both compounds **1a** and **1d** on diphenolase activity of tyrosinase is reversible.

### 3.4. *p*K*_a_ values and copper(II) affinity constant of 1n*

**Figure 5.**
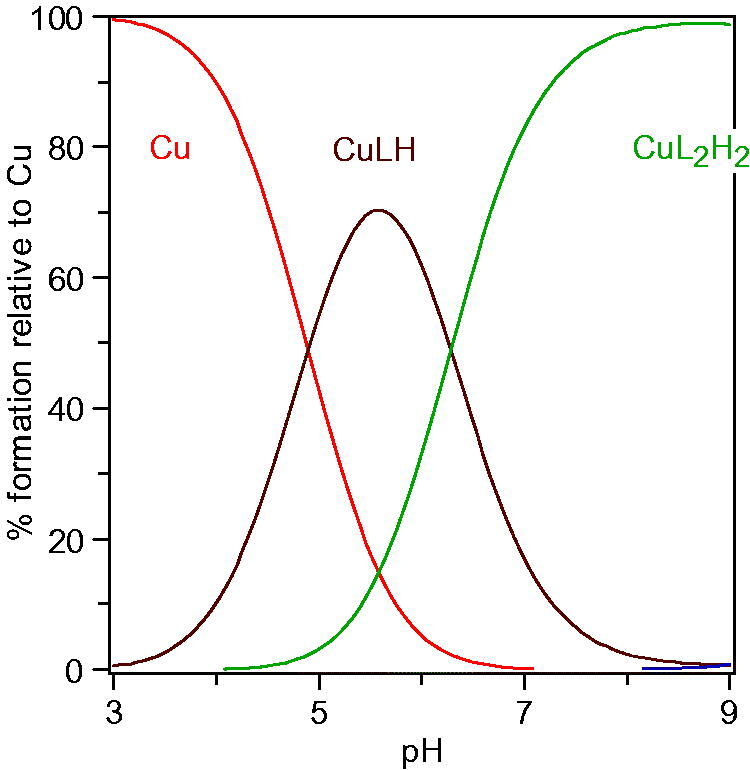
Speciation plots of copper ion in the presence of **1n**.

**Scheme 2.**
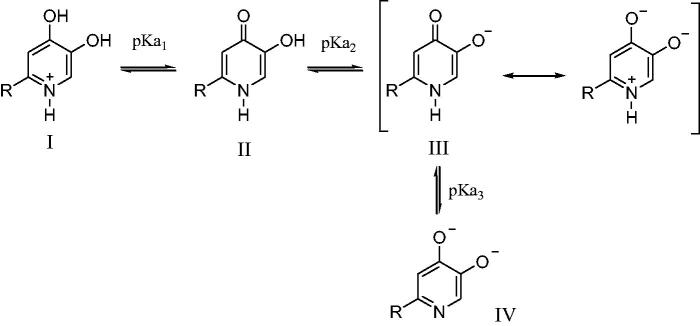
Proton equilibria and resonance structure of **1n**.

It is well known that the main inhibitory mechanism of kojic acid on tyrosinase involves chelation of copper in the active site in tyrosinase. Thus, for the purpose of exploring the inhibitory mechanism of compounds **1** on tyrosinase, the p*Ka* values of **1n** and its affinity for copper(II) were determined. The proton equilibria of **1n** are presented in Scheme 2. Using the spectrophotometric titration method, the three p*K*_a_ values of **1n** obtained from nonlinear least-squares regression analysis were found to be 3.3, 8.8, and 12.0, which correspond to the 4-oxo functional group, the 5-hydroxyl group, and 1-NH, respectively. As a bidentate, **1n** can form two species of copper complexes, CuLH and CuL_2_H_2_ (assuming 1-NH group does not deprotonate when forming the complexes with copper). The distribution of these two complexes varies with the pH (Figure 5). The log stability constants of these two complexes (CuLH and CuL_2_H_2_), log β_1_ and log β_2_, were determined to be 20.9 and 40.4, respectively. The pCu^2+^ value is a more suitable parameter to reflect the copper affinity of ligands, which is measured under the conditions of [Cu]_total_=10^−6^M, [L]_total_=10^−5^M, pH 7.4. The pCu^2^^+^ value of **1n** was calculated from the p*K*_a_ values and the log stability constants of its copper complexes using HYSS programme, being 9.9, which is greater than that of kojic acid (pCu^2+^=7.3)^29^. Thus, copper chelation is undoubtedly an important inhibitory mechanism on tyrosinase.

### 3.5. Molecular docking study

**Figure 6.**
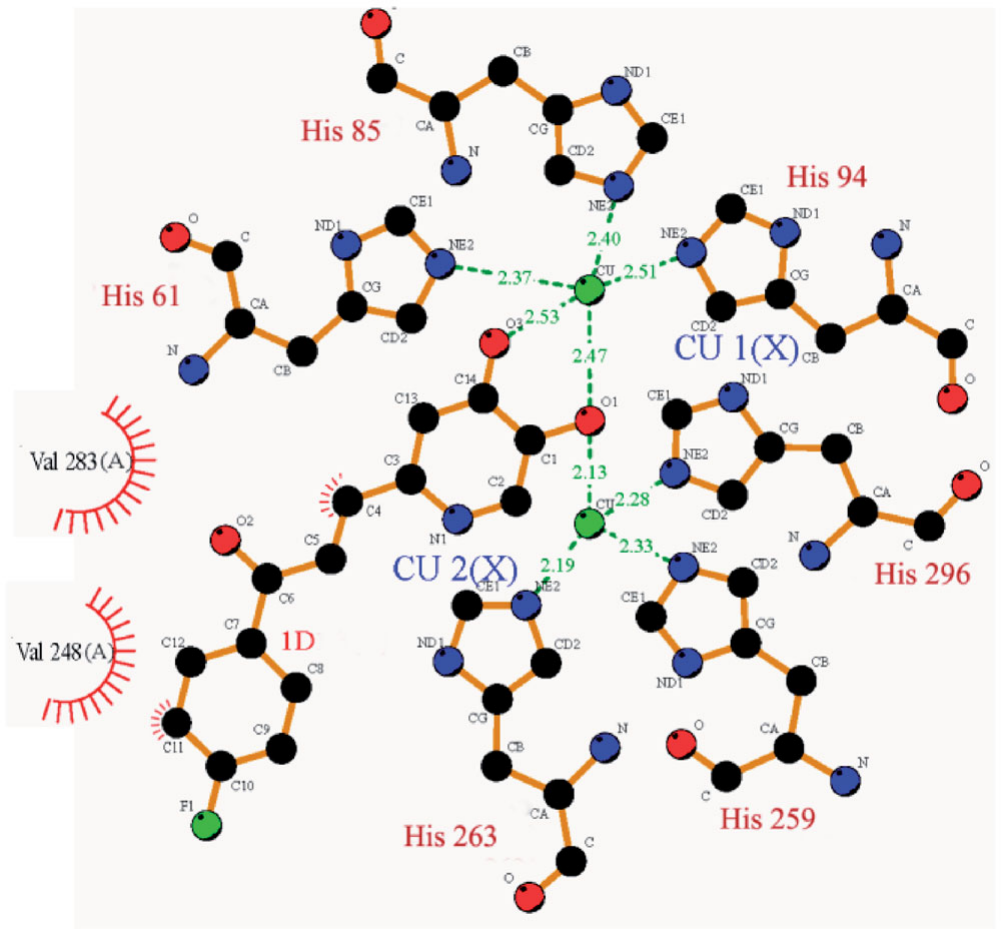
Configuration for the interaction of compound **1d** with tyrosinase.

In order to understand the interaction mode of inhibitor binding to tyrosinase, the molecular docking of compound **1d** to *Agaricus bisporus* tyrosinase was performed. As shown in Figure 6, compound **1d** binds to the active site of tyrosinase at the bottom of hydrophobic cavity of the receptor. The 4-oxy is located between Cu1 and Cu2 with a distance of 2.47 and 2.13 Å, respectively. The distance between Cu1 and the oxygen in 5-hydroxy group is 2.53 Å. Thus, the 4-oxy and 5-hydroxy group on the pyridinone ring can coordinate with Cu1, forming a 5-membered chelating ring; the 4-oxy can also coordinate with Cu2. Compound **1d** interacts with the side chain of Val283 and Val 248 via hydrophobic contacts, and interacts with the copper ligands His 61 and His263 via pi–pi stack, indicating the formation of a stable conformation.

## 4. Conclusions

Tyrosinase is an essential enzyme in melanogenesis metabolic process in microorganisms, plants, and animals, and thus is an attractive target for the discovery of novel anti-tyrosinase agents. In the present study, we have synthesised and screened a series of new chalcone analogues containing hydroxypyridinone moiety. Using enzyme inhibition assays, we have identified compound **1a, 1d,** and **1n** as potential lead molecules. The inhibitory effect of compounds **1a** and **1d** on diphenolase activity of mushroom tyrosinase activity is reversible. These findings support the previous proposal that kojic acid modification is a promising strategy for developing new potential tyrosinase inhibitors^30^^,^^31^

## Supplementary Material

Supplemental Material
