# Supplementary material for: Functionality study of chalcone-hydroxypyridinone hybrids as tyrosinase inhibitors and influence on anti-tyrosinase activity
Source: J Enzyme Inhib Med Chem. 2020 Aug 4;35(1):1562–7. doi: 10.1080/14756366.2020.1801669 (PMC7470021; doi:10.1080/14756366.2020.1801669)
Supplement: Supplemental Material [file IENZ_A_1801669_SM1632.pdf]

Supplementary Materials

Functionality study of chalcone-hydroxypyridinone hybrids as tyrosinase inhibitors and influence on anti-tyrosinase activity

L. Ravithej Singh,[a] Yu-Lin Chen,[b] Yuan-Yuan Xie,[c] Wei Xia,[a] Xin-Wen Gong,[a] Robert C. Hider,[b] and Tao Zhou*,[a]

[a] *School of Food Science and Biotechnology, Zhejiang Gongshang University, 18 Xuezheng Street, Xiasha, Hangzhou, Zhejiang 310018, P. R. China.*

[b] *Division of Pharmaceutical Science, King's College London, Franklin-Wilkins Building, 150 Stamford Street, London, SE1 9NH, UK*

[c] *College of Pharmaceutical Sciences, Zhejiang University of Technology, Hangzhou, 310014, P. R. China*

*Correspondence.

Tao Zhou, School of Food Science and Biotechnology, Zhejiang Gongshang University, 18 Xuezheng Street, Xiasha, Hangzhou, Zhejiang 310018, China. E-mail address: taozhou@zjgsu.edu.cn.

*General procedure for the synthesis of compounds **1a-1o***

Potassium tert-butoxide (0.79 mmol) was added to a stirred solution of the requisite phosphonate (**7a-7d**, 0.79 mmol) in THF (10 mL) at room temperature and stirred for 15 min. Aldehyde (**4a-4e**, 0.87 mmol) dissolved in THF (5 mL) was added dropwise via a syringe at 0-5 °C and the reaction mixture stirred for 2 h. The reaction was quenched with methanol (4 mL) and extracted with EtOAc (3 × 10 mL). The combined organic layers were then washed with brine (15 mL), dried and concentrated in vacuo to give a solid residue, which was dissolved in CH_2Cl_2 (5 mL).

The resulting solution was cooled in an ice-bath to 0-5 °C, BBr_3 (1.0 M in CH_2Cl_2, 5 mL) was added dropwise. After stirring at 0-5 °C for 50 min, the mixture was quenched with MeOH, diluted with water and extracted with CH_2Cl_2. The combined organic extracts were dried over anhydrous Na_2SO_4 and evaporated. Column chromatography of the residue over silica gel, using 1:30 MeOH-CH_2Cl_2, gave final compounds (**1a-1o**) as a yellow solid.

*(E)-2-(3-(4-fluorophenyl)-3-oxoprop-1-en-1-yl)-5-hydroxy-1-methylpyridin-4(1H)-one (**1a**)*. Yield: 71 %, ^{1}H NMR (400 MHz, DMSO-d_6) δ 8.30 (d, *J* = 5.5 Hz, 1H), 8.28 (d, *J* = 5.5 Hz, 1H), 8.27 (s, 1H), 8.08 (d, *J* = 15.4 Hz, 1H), 7.75 (d, *J* = 15.4 Hz, 2H), 7.45 (t, *J* = 8.9 Hz, 2H), 4.13 (s, 3H); ^{13}C NMR (101 MHz, DMSO-d_6) δ 187.15, 166.91, 164.39, 159.58, 145.66, 143.22, 133.43, 132.22, 130.98, 116.38, 116.16, 112.54, 44.37; HRMS (ESI): calcd for $C_{15}H_{13}FNO_3$ $[M + H]^+$ 274.0874, found 274.0880.

*(E)-5-hydroxy-2-(3-(2-hydroxy-4-methoxyphenyl)-3-oxoprop-1-en-1-yl)-1-methylpyridin-4(1H)-one (**1b**)*. Yield: 69 %, ^{1}H NMR (400 MHz, DMSO) δ 8.48 (s, 1H), 8.31 (s, 1H), 8.15 (d, J = 15.2 Hz, 1H), 7.80 (m, 2H), 6.76 (s, 1H), 4.15 (s, 3H), 3.95 (s, 3H); ^{13}C NMR (101 MHz, DMSO) δ 189.41, 164.35, 161.90, 159.49, 145.61, 142.87, 134.65, 133.30, 131.93, 130.94, 115.14, 112.62, 101.39, 101.24, 57.10, 44.22; HRMS (ESI): calcd for $C_{16}H_{15}NO_5$ $[M + H]^+$ 302.1023, found 302.1026.

*(E)-2-(3-(2,4-dimethoxyphenyl)-3-oxoprop-1-en-1-yl)-5-hydroxy-4H-pyran-4-one (**1c**)*. Yield: 65 %, ^{1}H NMR (400 MHz, DMSO) δ 8.13 (s, 1H), 8.07 (d, *J* = 9.0 Hz, 1H), 7.95 (d, *J* = 13.4 Hz, 1H), 7.45 (d, *J* = 15.4 Hz, 1H), 6.97 (s, 1H), 6.58 (d, *J* = 9.3 Hz, 1H), 6.40 (d, *J* = 8.5 Hz, 1H), 3.85 (s, 3H), 3.75 (s, 3H). HRMS (ESI): calcd for $C_{16}H_{14}O_6$ $[M + H]^+$ 303.0863, found 303.0859.

*(E)-2-(3-(4-fluorophenyl)-3-oxoprop-1-en-1-yl)-5-hydroxypyridin-4(1H)-one (**1d**)*. Yield: 66 %, ^{1}H NMR (400 MHz, DMSO) δ 8.26 (td, J = 8.8, 3.9 Hz, 3H), 8.18 (s,

1H), 7.80 (s, 1H), 7.67 (d, *J* = 15.7 Hz, 1H), 7.47 (t, *J* = 8.8 Hz, 2H); ^{13}C NMR (101 MHz, DMSO) δ 186.98, 166.86, 164.34, 160.77, 145.95, 140.62, 133.45, 131.97, 127.84, 116.36, 116.15, 112.48; HRMS (ESI) calcd for $C_{14}H_{10}FNO_3$ $[M + H]^+$ 260.0717, found 260.0721.

(E)-1-ethyl-5-hydroxy-2-(3-oxo-3-phenylprop-1-en-1-yl)pyridin-4(1H)-one ***(1e)***. Yield: 61 %, ^{1}H NMR (600 MHz, DMSO) δ 8.32 (s, 1H), 8.20 – 8.18 (m, 3H), 8.11 (d, *J* = 15.2 Hz, 1H), 7.77 (m, 2H), 7.62 (m, 2H), 4.53 (q, 7.2 Hz, 2H), 1.38 (t, *J* = 7.2 Hz, 3H); ^{13}C NMR (101 MHz, DMSO) δ 188.38, 159.85, 145.91, 142.40, 136.43, 134.07, 131.99, 131.68, 131.56, 129.03, 128.93, 113.04, 51.73, 15.81; HRMS (ESI): calcd for $C_{16}H_{15}NO_3$ $[M + H]^+$ 270.1125, found 270.1129.

(E)-2-(3-(3,4-dihydroxyphenyl)-3-oxoprop-1-en-1-yl)-1-ethyl-5-hydroxypyridin-4(1H) -one ***(1f)***. Yield: 62 %, ^{1}H NMR (400 MHz, DMSO) δ 8.32 (s, 1H), 8.00 (d, *J* = 15.3 Hz, 1H), 7.73 (s, 1H), 7.70 – 7.64 (m, 2H), 7.53 (d, *J* = 2.9 Hz, 1H), 6.89 (d, *J* = 8.2 Hz, 1H), 4.51 (q, *J* = 7.2 Hz, 2H), 1.37 (t, *J* = 7.2 Hz, 3H); ^{13}C NMR (101 MHz, DMSO) δ 186.15, 160.07, 152.01, 145.86, 142.77, 131.99, 131.76, 130.34, 128.61, 123.14, 115.50, 115.27, 112.79, 51.68, 15.81. HRMS (ESI): calcd for $C_{16}H_{15}NO_5$ $[M + H]^+$ 302.1023, found 302.1028.

(E)-1-ethyl-5-hydroxy-2-(3-(2-hydroxy-4-methoxyphenyl)-3-oxoprop-1-en-1-yl)pyridin-4(1H)-one ***(1g)***. Yield: 58 %, ^{1}H NMR (400 MHz, DMSO) δ 8.46 (s, 1H), 8.36 (s, 1H), 8.16 (d, *J* = 15.1 Hz, 1H), 7.82-7.79 (m, 3H), 6.76 (s, 1H), 4.54 (q, *J* = 7.2 Hz, 2H), 3.95 (s, 3H), 1.38 (t, *J* = 7.2 Hz, 3H); ^{13}C NMR (101 MHz, DMSO) δ 189.48, 164.35, 161.98, 159.68, 146.02, 142.22, 134.75, 132.23, 131.81, 131.47, 115.26, 113.20, 101.46, 101.31, 57.19, 51.90, 15.91. HRMS (ESI): calcd for $C_{17}H_{17}NO_5$ $[M + H]^+$ 316.1179, found 316.1334.

(E)-1-ethyl-2-(3-(4-fluorophenyl)-3-oxoprop-1-en-1-yl)-5-hydroxypyridin-4(1H)-one ***(1h)***. Yield: 65 %, ^{1}H NMR (400 MHz, DMSO) δ 8.29 (m, 3H), 8.10 (d, *J* = 15.3 Hz,

1H), 7.78 (d, *J* = 15.3 Hz, 1H), 7.73 (s, 1H), 7.45 (t, *J* = 8.8 Hz, 2H), 4.51 (q, *J* = 7.3 Hz, 2H), 1.37 (t, *J* = 7.2 Hz, 3H); ^{13}C NMR (101 MHz, DMSO) δ 187.09, 166.91, 164.39, 159.87, 146.05, 142.42, 133.28, 132.25, 132.15, 131.75, 116.14, 113.08, 51.90, 15.92; HRMS (ESI): calcd for $C_{16}H_{14}FNO_3$ $[M + H]^+$ 288.1030, found 288.1037.

(E)-5-hydroxy-1-methyl-2-(3-oxo-3-phenylprop-1-en-1-yl)pyridin-4(1H)-one ***(1i)***. Yield: 68 %, ^{1}H NMR (400 MHz, DMSO) δ 8.31 (s, 1H), 8.21 – 8.17 (m, 2H), 8.08 (d, *J* = 15.4 Hz, 1H), 7.81 (s, 1H), 7.78 – 7.71 (m, 2H), 7.62 (t, *J* = 7.8 Hz, 2H), 4.15 (s, 3H); ^{13}C NMR (101 MHz, DMSO) δ 196.45, 160.12, 147.59, 145.57, 143.76, 136.50, 134.59, 134.09, 129.07, 128.93, 128.04, 112.77, 43.96. HRMS (ESI): calcd for $C_{15}H_{13}NO_3$ $[M + H]^+$ 256.0968, found 256.0964.

(E)-1-butyl-2-(3-(3,4-dihydroxyphenyl)-3-oxoprop-1-en-1-yl)-5-hydroxypyridin-4(1H)-one ***(1j)***. Yield: 56 %, ^{1}H NMR (400 MHz, DMSO) δ 8.35 (s, 1H), 8.00 (d, J = 15.2 Hz, 1H), 7.77 (s, 1H), 7.69 (d, J = 15.2 Hz, 1H), 7.66 (dd, J = 8.4, 2.1 Hz, 1H), 7.53 (d, J = 2.1 Hz, 1H), 6.90 (d, J = 8.3 Hz, 1H), 4.50 (t, J = 7.4 Hz, 2H), 1.74 – 1.67 (m, 2H), 1.33 – 1.26 (m, 2H), 0.89 (t, J = 7.4 Hz, 3H). HRMS (ESI) calcd for $C_{18}H_{19}NO_5$ $[M + H]^+$ 330.1336, found 330.1335.

(E)-1-butyl-5-hydroxy-2-(3-(2-hydroxy-4-methoxyphenyl)-3-oxoprop-1-en-1-yl)pyridin-4(1H)-one ***(1k)***. Yield: 63 %, ^{1}H NMR (400 MHz, DMSO) δ 8.47 (s, 1H), 8.31 (s, 1H), 8.15 (d, J = 15.1 Hz, 1H), 7.86 – 7.74 (m, 3H), 6.76 (s, 1H), 4.52 – 4.49 (m, 2H), 3.95 (s, 3H), 1.76 – 1.67 (m, 2H), 1.30 (dq, *J* = 14.5, 7.2 Hz, 2H), 0.90 (t, *J* = 7.3 Hz, 3H). HRMS (ESI): calcd for $C_{19}H_{21}NO_5$ $[M + H]^+$ 344.1492, found 344.1500.

(E)-1-butyl-2-(3-(4-fluorophenyl)-3-oxoprop-1-en-1-yl)-5-hydroxypyridin-4(1H)-one ***(1l)***. Yield: 55 %, ^{1}H NMR (400 MHz, DMSO) δ 8.29 (dd, *J* = 9.5, 6.2 Hz, 3H), 8.10 (d, *J* = 15.2 Hz, 1H), 7.79 (d, *J* = 15.3 Hz, 1H), 7.76 (s, 1H), 7.45 (t, *J* = 8.8 Hz, 2H), 4.49 (t, *J* = 7.5 Hz, 2H), 1.74 – 1.68 (m, 2H), 1.34 – 1.26 (m, 2H), 0.89 (t, *J* = 7.4 Hz,

3H); ^{13}C NMR (101 MHz, DMSO-d6) δ 187.12, 166.92, 164.40, 159.86, 145.87, 142.58, 133.27, 132.26, 131.87, 116.37, 116.15, 113.09, 56.02, 32.23, 18.93, 13.46; HRMS (ESI): calcd for $C_{18}H_{18}FNO_3$ $[M + H]^+$ 316.1343, found 316.1343.

(E)-5-hydroxy-2-(3-oxo-3-phenylprop-1-en-1-yl)pyridin-4(1H)-one ***(1m)***. Yield: 72 %, ^1H NMR (400 MHz, DMSO) δ 8.27 (d, J = 15.8 Hz, 1H), 8.20 (s, 1H), 8.18 – 8.16 (m, 2H), 7.84 (s, 1H), 7.74 (t, J = 7.4 Hz, 1H), 7.68 (d, J = 15.8 Hz, 1H), 7.62 (t, J = 7.7 Hz, 2H); ^{13}C NMR (101 MHz, DMSO) δ 188.40, 160.76, 145.87, 140.55, 136.65, 134.10, 133.24, 131.60, 129.13, 128.91, 128.34, 112.67. HRMS (ESI): calcd for $C_{14}H_{11}NO_3$ $[M + H]^+$ 242.0812, found 242.0823.

(E)-2-(3-(3,4-dimethoxyphenyl)-3-oxoprop-1-en-1-yl)-5-hydroxypyridin-4(1H)-one ***(1n)***. Yield: 63 %, ^1H NMR (400 MHz, DMSO) δ 8.28 (d, *J* = 15.7 Hz, 1H), 8.16 (s, 1H), 7.93 (dd, *J* = 8.5, 1.9 Hz, 1H), 7.80 (s, 1H), 7.64 (d, *J* = 15.7 Hz, 1H), 7.61 (d, *J* = 1.9 Hz, 1H), 7.17 (d, *J* = 8.5 Hz, 1H), 3.89 (s, 3H), 3.87 (s, 3H); ^{13}C NMR (101 MHz, DMSO) δ 186.29, 160.82, 154.00, 149.03, 145.73, 140.77, 132.61, 129.65, 128.64, 128.26, 127.54, 124.16, 112.62, 111.12, 56.00, 55.80. HRMS (ESI): calcd for $C_{16}H_{15}NO_5$ $[M + H]^+$ 302.1023, found 302.1046.

(E)-5-hydroxy-2-(3-(2-hydroxy-4-methoxyphenyl)-3-oxoprop-1-en-1-yl)pyridin-4(1H) -one ***(1o)***. Yield: 60 %, ^1H NMR (400 MHz, DMSO) δ 8.45 (s, 1H), 8.26 (d, *J* = 15.6 Hz, 1H), 8.22 – 8.17 (m, 2H), 7.86 (s, 1H), 7.72 (d, *J* = 15.7 Hz, 1H), 6.76 (s, 1H), 3.95 (s, 3H); ^{13}C NMR (101 MHz, DMSO) δ 189.66, 164.71, 162.04, 160.72, 145.99, 140.44, 134.48, 133.49, 128.09, 127.78, 115.03, 112.60, 101.42, 101.32, 57.19. HRMS (ESI): calcd for $C_{15}H_{13}NO_5$ $[M + H]^+$ 288.0866, found 288.1258.
